# RNA modifications: emerging players in the regulation of reproduction and development: Function of RNA modifications in the reproduction and development

**DOI:** 10.3724/abbs.2024201

**Published:** 2024-11-21

**Authors:** Junfei Wen, Qifan Zhu, Yong Liu, Lan-Tao Gou

**Affiliations:** 1 Key Laboratory of RNA Innovation Science and Engineering Shanghai Key Laboratory of Molecular Andrology CAS Center for Excellence in Molecular. Cell Science Shanghai Institute of Biochemistry and Cell Biology Chinese Academy of Sciences Shanghai 200031 China; 2 University of Chinese Academy of Sciences Beijing 100049 China; 3 Anhui Province Key Laboratory of Embryo Development and Reproductive Regulation School of Biological and Food Engineering Fuyang Normal University Fuyang 236037 China

**Keywords:** RNA modification, reproduction, epitranscriptomic, mammalian development, developmental disease

## Abstract

The intricate world of RNA modifications, collectively termed the epitranscriptome, covers over 170 identified modifications and impacts RNA metabolism and, consequently, almost all biological processes. In this review, we focus on the regulatory roles and biological functions of a panel of dominant RNA modifications (including m ^6^A, m ^5^C, Ψ, ac ^4^C, m ^1^A, and m ^7^G) on three RNA types—mRNA, tRNA, and rRNA—in mammalian development, particularly in the context of reproduction as well as embryonic development. We discuss in detail how those modifications, along with their regulatory proteins, affect RNA processing, structure, localization, stability, and translation efficiency. We also highlight the associations among dysfunctions in RNA modification-related proteins, abnormal modification deposition and various diseases, emphasizing the roles of RNA modifications in critical developmental processes such as stem cell self-renewal and cell fate transition. Elucidating the molecular mechanisms by which RNA modifications influence diverse developmental processes holds promise for developing innovative strategies to manage developmental disorders. Finally, we outline several unexplored areas in the field of RNA modification that warrant further investigation.

## Introduction

RNA building blocks, *i.e*., A, U, C and G ribonucleotides, naturally undergo various chemical modifications. First reported in the 1950s [ 1– 4], more than 170 modifications have been identified to date, and most types of RNA, if not all, have been observed with modifications [5]. These modifications, collectively known as the epitranscriptome, alter the inherent features of single nucleotides and greatly increase the information-encoding capacity of RNA.

RNA modification plays a central role in the regulation of RNA metabolism. The regulatory functions of modifications are best studied for three types of RNA that are indispensable for translation—transfer (tRNA), ribosomal (rRNA), and messenger RNA (mRNA). Modifications of these RNAs influence nearly all steps of RNA metabolism, such as RNA processing, RNA structure, cellular localization, stability, and translation efficiency, functioning as key connections between gene transcription and protein synthesis.

Using high-throughput sequencing methods, several RNA modifications, such as *N* ^6^-methyladenosine (m ^6^A) and 5-methylcytidine (m ^5^C), have been mapped transcriptome-wide in various samples. With the development of low-input methods, the landscape of those RNA modifications has recently been revealed in rare cell populations, such as oocytes and early embryos. The spatiotemporal dynamics of RNA modifications across different biological contexts have significantly enriched our comprehension of their cellular roles. During development, cell fates are tightly regulated in each cell and coordinated within a cell population. In response to internal and/or external stimuli, cells make decisions to self-renew, proliferate or differentiate, which often requires prompt rewiring of the proteome before a new transcriptional profile can be fully established. RNA modifications, which are deposited in advance or swiftly altered in response to cues, may serve as a regulatory mechanism enabling rapid proteomic adaptation [6]. As this tight modulation of the proteome is pivotal for cell state transition, it is not surprising that dysfunction of RNA modification-related proteins (i.e., writers, erasers and readers) and abnormal deposition of RNA modifications have been linked to infertility [ 7 – 13], developmental disorders [ 14– 16], and various diseases [17].

In this review, we describe the functions of modifications detected in RNA, especially mRNAs that play a vital role in RNA metabolism, with a focus on the modifications whose functions in the mammalian reproductive system and embryonic development have been investigated or inferred. We discuss how their roles in RNA metabolism in turn affect stem cell self-renewal, cell fate transition and other developmental processes. Currently, the functions of m ^6^A and m ^5^C on mRNAs are relatively well studied and are therefore discussed in depth in this review. A few more modifications, such as Ψ and m ^1^A, have only been recently detected in mRNAs. Although the functions of these modifications on mRNAs remain largely undetermined, we appreciate the diversity of RNA modifications and therefore briefly discuss their potential roles in RNA metabolism. Further research into the specific mechanisms by which RNA modifications influence various developmental processes could reveal new strategies for managing developmental disorders and improving reproductive health.

## Roles of RNA Modifications and Their Regulators in RNA Metabolism

**Figure 1:**
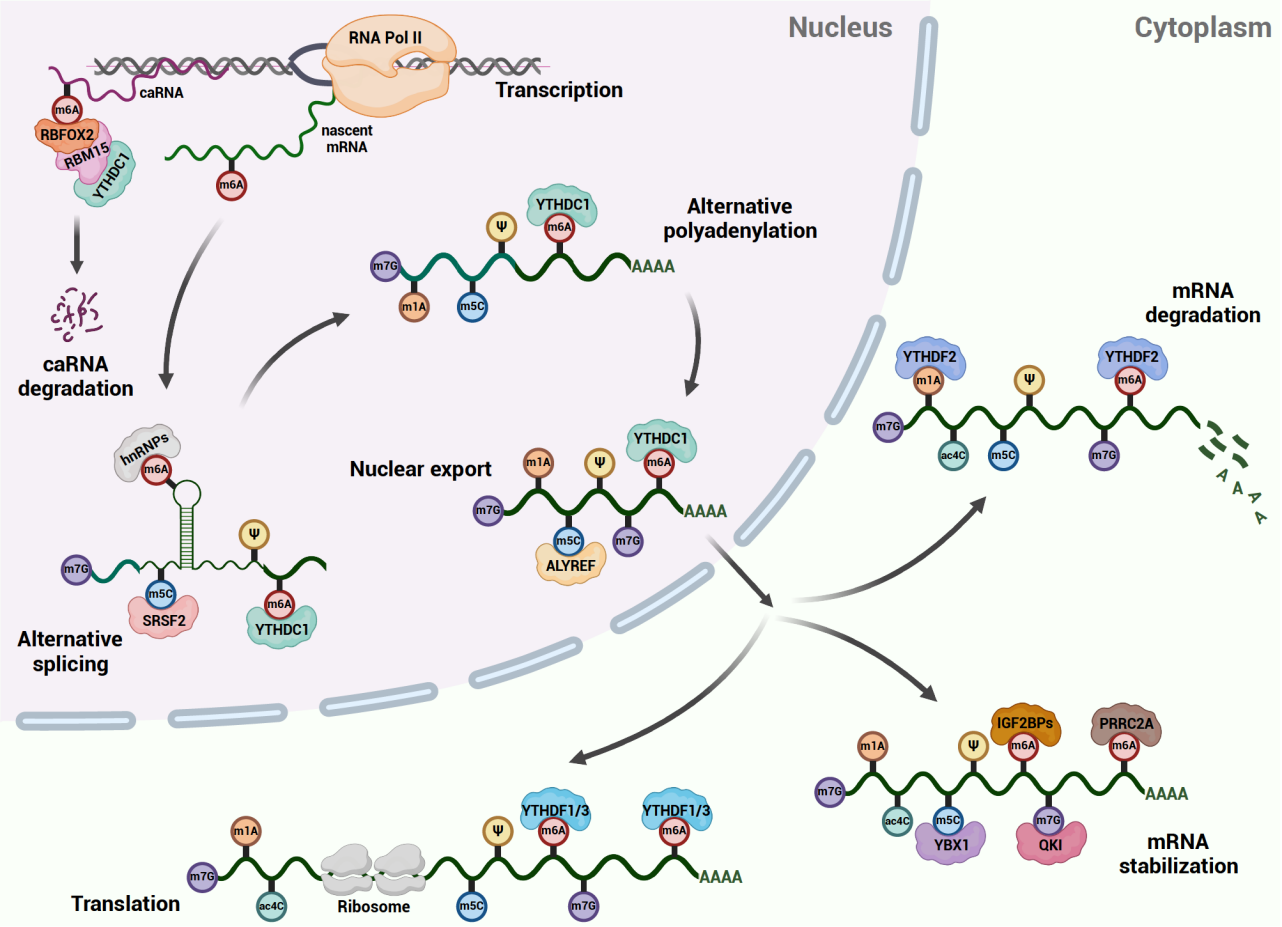
Figure 1 Overview of RNA modifications in RNA metabolism regulation The life of RNA undergoes fine-tuned complex RNA metabolism starting with transcription and alternative splicing in nucleus, then export from nucleus to cytoplasm, and finally translated, stabilized, or degraded in the cytoplasm. RNA modifications are shown to influence nearly all steps of RNA metabolism.

The dynamic nature of RNA modifications allows for the fine-tuning of RNA metabolism, which is essential for the successful development of an organism from a single cell to a complex multicellular entity ( Figure 1). The regulation of RNA modification is mediated by a complex process involving various proteins, often referred to as “writers”, “erasers” and “readers”. In this section, we describe the regulators and molecular functions of RNA modification in refining RNA metabolism ( Table 1).

**Table:** **Table 1** Regulators of RNA modifications

Modification	Regulators			Localization	Currently known targets and/or functions	Related mammalian developmental disorders/events when the regulator is dysfunctional or dysregulated
m ^6^A	Writers	METTL3	Catalytic protein	Nucleus; cytoplasm [ 18– 20]	RNA polymerase II-derived transcripts [ 21– 24]	Male infertility [ 11, 12]; female infertility [ 25, 26]; defects in cerebellum development [ 27, 28]; defects in retionogenesis [29]; sress- and age-related cardiac failure [30]; perinatal hepatocyte injury [31]; defective liver glycogenesis [32]; impaired osteogenic differentiation potential of mesenchymal stem cells (MSCs) differentiation into osteoblasts [33]
		METTL14	Adaptor	Nucleus		Male infertility [ 11, 12]; dysregulated cell cycle in cortical neurogenesis [34]; defects in neural stem cells (NSCs) self-renewal [35]; disrupted cell cycle and differentiation in retinal progenitors [36]
		WTAP	Cofactor	Nucleus		Dilated cardiomyopathy [37]
		RBM15/15B				n/a
		ZC3H13				n/a
		VIRMA (KIAA1429)				Female infertility [38]
		DDX21				n/a
		METTL4	Catalytic protein	Nucleus	U2 snRNA [ 39, 40]	n/a
		METTL16	Catalytic protein	Nucleus	U6 snRNA; MAT2A mRNA; various ncRNA; pre-mRNA [ 41– 43]	Male infertility [44]; embryonic arrest around the time of implantation due to transcriptional reduction of its target MAT2A [45]
		METTL5	Catalytic protein	Nucleus	rRNA [ 46– 48]	Abnormal myelination and intelligence [49]
		TRMT112	Cofactor	Nucleus; perinuclear cytoplasm	n/a
		ZCCHC4	Cofactor	Nucleus; cytoplasm	n/a
	Erasers	FTO	Catalytic protein	Nucleus	mRNA; snRNA [50]	Age-dependent male subfertility [51]; female infertility [52]; defective myogenic differentiation [53]
		ALKBH5	Catalytic protein	Nucleus; cytoplasm	mRNA [ 54, 55]	Male infertility [54]; female infertility [56]; abnormal cell proliferation and differentiation within the cerebellum under hypobaric hypoxia [29]
		RBM33	Cofactor	Nucleus		n/a
		ALKBH3	Catalytic protein	Nucleus	tRNA [57]	n/a
	Readers	YTHDC1	Direct binding	Nucleus	Regulates mRNA splicing, polyadenylation and nuclear export [ 58– 60]; represses retrotransposons [61]	Male infertility [59]; female infertility [59]; dilated cardiomyopathy [62]
		YTHDC2		Nucleus; Cytoplasm	Promotes mRNA translation and then decay [9]	Male infertility [ 9, 13]; female infertility [9]
		YTHDF1		Cytoplasm	Promotes mRNA translation [63]	Viable and fertile [26]; learning and memory defects [64]
		YTHDF2		Cytoplasm	Promotes mRNA decay [65]	Male infertility [66]; female infertility [67]; preweaning lethality with incomplete penetrance due to compromised neural development [67]
		YTHDF3		Cytoplasm	Promotes mRNA translation and then decay [68]	Viable and fertile [26]
		ElF3	GG(m ^6^A)CU motif-based	Cytoplasm	Promotes translation in associate with YTHDF1 [ 63, 69]	n/a
		hnRNPs	m6A structural switch-based ( *i*. *e*., indirect binding)	Nucleus	Regulates RNA splicing [70]	n/a
		IGF2BP1/IGF2BP2/IGF2BP3	GG(m ^6^A)CU motif-based	Nucleus; cytoplasm	Promotes mRNA stability and translation [71]	n/a
		FMR1 (FMRP)	GG(m ^6^A)CU motif-based	Nucleus; cytoplasm	Facilitates mRNA nuclear export [72]; modulates mRNA stability [73]; represses the translation of its targets [74]	Dysregulated cell cycle in neurogenesis [72]
		PRRC2A	GG(m ^6^A)CU motif-based	Cytoplasm	Promotes RNA stability [75]	Male infertility [76]; defects in oligodendrocyte specification [75]
		CNBP	Uncertain	Nucleus; cytoplasm	Promotes mRNA stability and translation [77]	n/a
		ELAVL1 (HUR)	Uncertain	Nucleus; cytoplasm	Uncertain	n/a
		RBFOX2	Uncertain	Nucleus	Promotes m ^6^A-dependent chromatin silencing [78]	n/a
m ^5^C	Writers	NSUN1	Catalytic protein	Nucleus	rRNA [ 79, 80]	n/a
		NSUN2		Nucleus	tRNA [81], mRNA [82], ncNRA [ 83, 84], viral RNA [85]	Male infertility [86]; severely defected short-term-memory [87]
		NSUN3		Mitochondria	mt-tRNA [ 16, 88]	Encephalomyopathy [ 16, 89]
		NSUN4		Mitochondria	mt-rRNA [ 90, 91]	n/a
		NSUN5		Nucleus	rRNA [ 92, 93]	Age-dependent female subfertility [94]; Williams-Beuren syndrome (WBS) [ 93, 95– 97]
		NSUN6		Cytoplasm	tRNA [98], mRNA [ 99, 100].	Locomotion and learning impairment [101]
		NSUN7		Uncertain	eRNA [102]	Male infertility [ 103, 104]
		DNMT2		Nucleus	tRNA [105], viral RNA [106]	Intergenerational epigenetic inheritance [105]
	Erasers	TET family	Catalytic protein	Nucleus	Retrotransposon RNA [107]	n/a
		ALKBH1		Cytoplasm; mitochondria	Cytoplasmic tRNA; mt-tRNA [108]	n/a
	Readers	ALYREF	Direct binding	Nucleus	Facilitates nuclear-cytoplasmic shuttling of mRNA [109]	n/a
		YBX1		Cytoplasm	Promotes mRNA stability [110]; represses mRNA translation [111]	n/a
		YTHDF2		Cytoplasm	Factilitates pre-rRNA processing [112]	n/a
m ^1^A	Writers	RRP8 (NML)	Catalytic protein	Nucleus	rRNA [113]	n/a
		TRMT6/61A		Nucleus; cytoplasm	tRNA, mRNA [ 114, 115]	n/a
		TRMT61B	Catalytic protein	Mitochondria	mt-rRNA, mt-tRNA [ 114, 116]	n/a
		TRMT10C	Catalytic protein	Mitochondria	mt-tRNA [117]	n/a
	Erasers	ALKBH1	catalytic	Cytoplasm	tRNA [118]	n/a
		ALKBH3		Nucleus; cytoplasm	tRNA [119], mRNA [120]	n/a
		ALKBH7		Mitochondria	mt-pretRNA and mt-dsRNA [121]	n/a
		FTO		Nucleus	tRNA [50]	n/a
	Readers	YTHDC1	Direct binding	Nucleus	Uncertain	n/a
		YTHDF1		Cytoplasm	Uncertain	n/a
		YTHDF2		Cytoplasm	Promotes mRNA destabilization [ 122, 123]	n/a
		YTHDF3		Cytoplasm	n/a
		TDP-43 (TARDBP)	Uncertain	Nucleus	Binding of TDP-43 to m ^1^A-containing CAG repeat RNA induces its sequestration into stress granules [124]	n/a
m ^7^G	Writers	METTL1-WDR4 complex	Catalytic complex	Nucleus	tRNA, mRNA, miRNA [ 125, 126]	Cardiac fibrosis [127]
		RNMT-RAM complex		Nucleus	m ^7^G-cap [ 128– 130]	n/a
		TGS1		Nucleus	TMG-cap [ 131, 132]	n/a
		WBSCR22-TRMT112 complex		Nucleus; perinuclear cytoplasm	rRNA	n/a
	Readers	Nuclear cap binding protein complex (CBC)	m ^7^G-cap	Nucleus	Facilitates the maturation [133] and export of mRNA [134]	n/a
		eIF4E	m ^7^G-cap	Nucleus; cytoplasm	Promotes translation initiation; facilitates processing and nuclear export of mRNA [ 135– 137]	n/a
		PARN	m ^7^G-cap		Promotes dedeadenylation [ 138, 139]	n/a
		Snuportin-1 (SNUPN)	TMG-cap	Nucleus; cytoplasm	Transports snRNAs from the cytoplasm to nucleus [ 140, 141]	n/a
		Exportin-1 (XPO1/CRM1)	TMG-cap	Nucleus; cytoplasm	Transports U3 snoRNA from Cajal bodies to nucleoli [142] and pri-miRNA to the cytoplasm [143]	n/a
		QKI	Internal m7G	Cytoplasm	Interacts with G3BP1 and represses translation by relocating targets into stress granule [144]	n/a
ac ^4^C	writers	NAT10	Catalytic protein	Nucleus (nucleoli)	tRNA [145], rRNA [ 146 , 147], mRNA [ 148– 150]	Male infertility [151]; female infertility [152]
Ψ	Writers	PUS1	Catalytic protein	Nucleus; mitochondria	mRNA [153], tRNA, mt-tRNA [154]	Mitochondrial myopathy with lactic acidosis and sideroblastic anemia (MLASA) [155]
		PUSL1		Mitochondria (inner membrane [156])	mRNA [157]	n/a
		PUS3		Nucleus; cytoplasm	mRNA, tRNA [157]	Intellectual disability [158]
		PUS7		Nucleus	mRNA, tRNA [157]	Intellectual disability and microcephaly [159]
		PUS7L		Nucleus (nucleoli; nuclear speckle)	mRNA, tRNA [157]	n/a
		PUS10		Nucleus (nucleoplasm and nuclear bodies); mitochondria	mRNA, tRNA [153]	n/a
		TRUB1		Nucleus; mitochondria	mRNA, tRNA, mt-tRNA [ 153, 157, 160]	n/a
		TRUB2		Mitochondria	mRNA, tRNA, mt-tRNA, mt-mRNA [ 153, 157, 161]	n/a
		DKC1		Nucleus	mRNA, rRNA, snoRNA, snRNA [ 154, 157]	Defective proliferation of hepatocytes [162]
		RPUSD2		Nucleus; mitochondria	mRNA [153]	n/a
		RPUSD3		Nucleus; mitochondria	mRNA, mt-mRNA [163]	n/a
		RPUSD4		Nucleus; mitochondria	mRNA, mt-rRNA [ 153, 163]	n/a

### *N* ^6^-methyladenosine (m ^6^A)

#### Writer

m ^6^A, one of the most abundant RNA modifications and probably the most studied one at present, was first identified in mammalian mRNAs in 1975 [164] and further detected in almost all RNA types [5]. The methyl group conjugated to the sixth nitrogen atom of the adenosine nucleobase ( *i*. *e*., *N* ^6^) has been found to be installed by several methyltransferases, frequently operating as protein complexes. This m ^6^A methyltransferase complex (MTC) for mRNA was characterized and partially purified in 1994 [165], with individual components subsequently identified. The core components of MTC, methyltransferase-like 3 (METTL3) [22] as the main catalytic subunit and methyltransferase-like 14 (METTL14) as the activator [ 23, 24], form a heterodimer that co-transcriptionally deposits m ^6^A within a conserved DRACH motif (D = A/G/U, R = A/G, and H = U/A/C) on mRNAs and other RNA polymerase II-derived transcripts, such as chromosome-associated regulatory RNA (carRNA) [21]. Wilms tumor 1-associated protein (WTAP) [166], first classified as a splicing factor, is another key subunit of the MTC that mediates the nuclear localization of METTL3-METTL14 complex. Additional subunits, such as vir-like m ^6^A methyltransferase associated (VIRMA or KIAA1429) [167] and zinc finger CCCH-type containing 13 (ZC3H13) [168], which are known to interact with WTAP, are also crucial for m ^6^A synthesis, as their ablation leads to considerable loss of m ^6^A in mammalian cells [169]. KIAA1429, which interacts with polyadenylation cleavage factors, was suggested to facilitate the preferential deposition of m ^6^A in the 3′UTR and near the stop codon [167]. However, recent studies argue that m ^6^A deposition is not selective. Instead, the scarcity of m ^6^A on the CDS and 5′UTR resulted from steric suppression by exon junction complexes (EJCs) [ 170– 172]. ZC3H13, like WTAP, is required for the nuclear localization of MTC [168] and promotes the interaction between WTAP and another MTC component, RNA binding motif protein 15/15B (RBM15/RBM15B) [173]. RBM15/15B is reported to recruit MTC to target transcripts such as XIST [174]. A recent study revealed that another cofactor, DDX21, anchors to the R-loop, recruits METTL3 and is therefore key to the co-transcriptional addition of m ^6^A to nascent RNAs [175]. In addition to the METTL3-METTL14 complex, three additional methyltransferases are responsible for m ^6^A deposition on other types of RNAs. METTL16 deposits m ^6^A on a single site of U6 small nuclear RNA (snRNA), a U6-like sequence within *MAT2A* mRNA [ 41, 42], non-coding RNAs (ncRNAs) and pre-mRNAs [43]. METTL4 forms m ^6^A on U2 snRNAs, regulating RNA splicing [ 39, 40]. Most m ^6^A is located in abundant ribosome RNA (rRNA), which is mediated by the METTL5-TRMT112 complex (18S rRNA) [ 46, 47 ] and zinc finger CCHC-type containing 4 (ZCCHC4) (28S rRNA) [48].

#### Reader

To regulate RNA metabolism, a variety of proteins can be specifically recruited to m ^6^A sites, facilitating the processing, transport, translation and stability of mRNAs. One major m ^6^A reader family is the IYT521-B homology (YTH) family, of which five members have been identified as m ^6^A readers, and the YTH domain is critical for the direct recognition of m ^6^A. The first reported m ^6^A-binding protein or reader, YTH *N* ^6^-methyladenosine RNA binding protein F2 (YTHDF2), was recovered from an *in vitro* m ^6^A RNA pull-down experiment. YTHDF2, which is present mainly in the cytoplasm, binds to m ^6^A-modified mRNAs and relocates them from the translatable pool to mRNA decay sites [65]. YTHDF1, another YTH-containing m ^6^A reader, promotes the translation of YTH-binding transcripts [63]. YTHDF3, which functions at an earlier time point in the RNA life cycle, can interact with YTHDF1, facilitating translation, and eventually YTHDF2, accelerating RNA decay [68]. YTHDC1, unlike the above cytoplasmic YTHDFs, is a nuclear m ^6^A reader that regulates mRNA polyadenylation and splicing by recruiting splicing factors [ 58, 59]. It also recruits nuclear export adaptors that regulate nuclear export [60]. Although predominantly localized in the nucleus, YTHDC1 exhibits dynamic subcellular localization by shuttling between the nucleus and cytoplasm [176]. This mobility enables YTHDC1 to play a crucial role in regulating the stability of MAT2A mRNA, which is modified by METTL16 [60]. Unlike YTHDC1, YTHDC2 mainly resides in the cytoplasm, where it likely engages with the translation and decay machinery to increase translation efficiency and reduce the mRNA levels of its targeted transcripts [9]. However, whether translational regulation by YTHDC2 is m ^6^A dependent remains to be confirmed [177].

YTH family members directly bind to m ^6^A sites through the YTH domain, whereas heterogeneous ribonucleoprotein particles (hnRNPs), a known RNA-binding protein (RBP) family, recognize the RNA structural changes induced by m ^6^A, *i*. *e*., indirect binding. This structural change induced by m ^6^A, also known as the m ^6^A structural switch, increases the accessibility of the adenosine site and adjacent regions, thereby increasing the binding affinity of hnRNPs. HNRNPA2B1 [70], HNRNPC [178], and HNRNPG [74] are examples of such indirect m ^6^A readers. As hnRNPs act as splicing regulators, their binding mediates the splicing of m ^6^A-labelled transcripts, including both mRNAs and primary miRNAs (pri-miRNAs) [70].

Insulin-like growth factor-2 mRNA-binding proteins (IGF2BPs) constitute another m ^6^A reader family. IGF2BPs can directly recognize m ^6^A within the consistent motif GG(m ^6^A)CU. The binding specificity is mediated via the K-homology (KH) domains of IGF2BPs. The binding of three IGF2BP family members (IGF2BP1/2/3) to m ^6^A-labelled mRNAs enhances their stability, promotes their storage under stress conditions, and facilitates translation after being shuttled to the cytoplasm [71].

Apart from the aforementioned three families, a few additional proteins are also suggested to have m ^6^A-binding ability. Eukaryotic initiation factor 3 (eIF3), a known translation initiation factor, is associated with the m ^6^A site in the 5′UTR and promotes translation in association with YTHDF1. Whether the m ^6^A binding of eIF3 is direct or YTHDF1-dependent remains controversial and may be context-dependent [ 63, 69]. Furthermore, a recent study using multiple assays, including structural data of m ^6^A-eIF2α interaction, did not support a translation-promoting role of the m ^6^A site in the 5′UTR, warranting further clarification of the role of eIF3 as a m ^6^A reader [179]. Proline-rich coiled-coil 2 A (PRRC2A), a m ^6^A reader identified in oligodendrocytes, binds to the consensus GG(m ^6^A)CU motif and promotes RNA stability [75]. The consensus GG(m ^6^A)CU motif is also targeted by another two m ^6^A readers, FMRP ( *FMR1*) [ 72– 74, 180, 181] and CNBP [77]. FMRP binds to ribosomes and is reported to facilitate nuclear export [72], modulate stability [73], and repress the translation of its targets [74]. The target selection of FMRP is preferential but not strictly dependent on m ^6^A [74]. CNBP, on the other hand, promotes stability and translation. However, the targets of CNBP and the mechanism by which it binds to m ^6^A sites may require further investigation. ELAVL1 (also known as HUR) has also been suggested to be strongly associated with m ^6^A probes in RNA affinity chromatography experiments, but whether and how it targets m ^6^A *in vivo* remain unknown [182]. RBFOX2, a well-studied splicing factor, has been recently suggested to preferentially recognize m ^6^A on chromatin-associated RNAs (caRNAs). By recruiting RBM15 and YTHDC1, RBFOX2 further promotes the methylation of promoter-associated RNAs and attracts polycomb repressive complex 2 (PRC2), resulting in m ^6^A-dependent, locus-selective chromatin silencing and transcription suppression [78].

#### Eraser

The discovery of erasers indicates that m ^6^A is a reversible RNA modification. To date, three m ^6^A demethylases responsible for the removal of m ^6^A from mRNAs, tRNAs and some ncRNAs have been reported. Fat mass and obesity-associated protein (FTO), the first m ^6^A eraser discovered, displays demethylation activity against m ^6^A in a pH-dependent manner according to *in vitro* enzymatic analysis. Knockdown of *FTO* in cell lines leads to a moderate increase in the global m ^6^A level [ 50, 183]. Later studies reported the demethylation activity of FTO toward another modification that is structurally similar to m ^6^A, *N* ^6^ ,2′- *O*-dimethyladenosine (m ^6^A _m_), on mRNAs and snRNAs to regulate mRNA stability [184] and snRNA processing [185]. m ^6^A erasure may have specific importance in specific tissues, such as the testes. The second identified demethylase, alkB homologue 5 (ALKBH5), whose knockout in mice results in no defects in general development or health except in spermatogenesis [ 186, 187]. A further study revealed that in spermatocytes and round spermatids, ALKBH5-mediated m ^6^A erasure regulates splicing and the production of longer 3′-UTR mRNAs [54]. In a recent study, a cofactor of ALKBH5, RBM33, was identified. RBM33, a m ^6^A reader, binds to m ^6^A via its RNA recognition motif (RRM) domain and enhances the affinity of ALKBH5 for m ^6^A as well as its demethylation activity, providing a regulatory mechanism of m ^6^A erasure for the first time [55]. *In vitro* assays indicate that another ALKBH family protein, ALKBH3, is also an eraser for m ^6^A on tRNA [57], although *in vivo* studies suggest that ALKBH3 mainly targets m ^1^A.

### 5-Methylcytidine (m ^5^C)

#### Writer

m ^5^C in RNA, i.e., methylation of cytosine residues at position 5, was identified in *Escherichia coli* in 1958 [1] and has been observed in various types of RNAs, including mRNAs, enhancer RNAs (eRNAs), miRNAs, tRNAs, rRNAs and small RNAs derived from tRNAs (tsRNAs) and rRNAs (rsRNAs). The NOL1/NOP2/sun domain-containing (NSUN) genes encode RNA methyltransferases that catalyze m ^5^C. Seven members (NSUN1–7) have been confirmed to have catalytic activity, each with different subcellular localizations and to target different types of RNA substrates.

NSUN1 mainly targets rRNAs. NSUN1, also known as p120 or NOP2, resides in nucleoli and modifies C4447 in human 28S rRNA and C2870 in yeast 25S rRNA [ 79, 80]. Interestingly, NSUN1 was also found to form a complex with BRD4 and Pol II in an m ^5^C-dependent manner, suggesting that it may be associated with m ^5^C on mRNA [188]. Additionally, NSUN1 has also been reported as an HIV-1 restriction factor. Its HIV-1 transcription-inhibiting function may be related to m ^5^C on an HIV-1 RNA hairpin, TAR [189].

NSUN2, which is localized in the nucleus of most cell types, has been reported to target various tRNAs, mRNAs and ncRNAs. For tRNAs, NSUN2 favors the C34, C40, C48, C49, and C50 sites in the variable loop, which contributes to tRNA stability and, in turn, protein synthesis [81]. NSUN2 also targets a selection of mRNAs, of which m ^5^C sites are usually found within CDS regions [82]. NSUN2-mediated m ^5^C may promote mRNA export [109]. In addition to tRNA and mRNA, vtRNA [ 83, 84 ], a type of sncRNA, and virus transcripts [85] are also targets of NSUN2. NSUN2 participates in stem cell self-renewal and differentiation [ 86, 190, 191] and is reportedly associated with neural tube and somite formation during early development, spermatogenesis and Dubowitz-like syndrome, a multisystem developmental disorder [ 192 , 193].

NSUN3 and NSUN4 catalyze the methylation of specific mitochondrial tRNAs and rRNAs, respectively. NSUN3 modifies C34 of mt-tRNA ^Met^, forming m ^5^C34 [ 16, 88 ], which is then oxidized by ALKBH1 (ABH1) to form 5-formylcytosine at position 34 (f ^5^C34) [194]. Position 34 in mt-tRNA ^Met^ is the first (wobble) position of the anticodon, the modifications of which often modulate codon recognition and translation accuracy. f ^5^C34 enables single mt-tRNA ^Met^ to recognize both the AUA and AUG codons [195]. Deficiency of NSUN3 has been linked to dysregulation or reduction of mitochondrial protein synthesis, which downstream mediates mouse embryonic stem cell (ESC) differentiation towards the mesoderm and endoderm [196]. NSUN4 modifies C911 in the 12S rRNA (m ^5^C911) of human and mouse mitochondria and forms a complex with MTERF4 to mediate mitoribosomal assembly [ 90, 91]. Its deletion therefore leads to the inhibition of mitochondrial translation and mitochondrial dysfunction.

The target of NSUN5 is rRNA, specifically C3782 in human 28S rRNA [92], C3438 in mouse 28S rRNA [93], C2268 in *Arabidopsis thaliana* 25S rRNA, and C2278 in yeast 25S rRNA [80]. Like NSUN1, NSUN5 is enriched in nucleoli. The deletion of NSUN5 leads to a global decrease in translation and is known to be associated with Williams-Beuren syndrome (WBS), a neurodevelopmental disorder [93]. In yeast, worms, and flies, NSUN5 has also been reported to affect lifespan and regulate stress responses [197].

NSUN6, a cytoplasmic protein, specifically targets C72 in human tRNA ^Cys^ and tRNA ^Thr^ [98]. This specific target recognition is mediated by both the protein domain of NSUN6 and the sequence features of its targeted tRNAs. An *in vitro* assay indicated that only well-folded, full-length tRNA substrates can be recognized and methylated by NSUN6. A few components of the tRNA sequence, including a CCA terminus, the discriminator base U73, two base pairs (2:71 and 3:70) in the acceptor region and another two (11:24 and 12:23) in the D-loop, are important for recognition by NSUN6 [198]. The CCA terminus is recognized by the PUA domain of NSUN6, the discriminator base U73 is bound by the RRM motif, and C72 is methylated by the catalytic core [199]. Intriguingly, recent studies have also reported that NSUN6 targets a selection of mRNAs via methylation-dependent individual-nucleotide resolution cross-linking and immunoprecipitation (miCLIP) [ 99, 100]. This mRNA-catalyzing activity of NSUN6 was further confirmed by the identification of a K159A/R181A variant that largely loses tRNA methylation but retains its ability to methylate mRNAs [100]. To date, two types of m ^5^C sites have been identified on mRNAs. Type I, which is mainly mediated by NSUN2, is predicted to reside at the 5′ end of putative hairpin structures, which are composed of a G-rich triplet motif. Type II proteins, which are mediated by NSUN6, are predicted to reside in the loop of putative hairpin structures within the 3′UTR, where a consensus motif (m ^5^C)UCCA is present. Proximity labelling of RNA indicated that transcripts containing type I m ^5^C are enriched for nuclear localization, whereas type II sites are enriched for the endoplasmic reticulum membrane (ERM) and outer mitochondrial membrane (OMM), suggesting differential functions of NSUN2- and NSUN6-mediated methylation [100] .

The last m ^5^C writer of the NSUN family, NSUN7, was reported to act on a unique type of ncRNA, enhancer RNAs (eRNAs). A group of eRNAs are selectively methylated by NSUN7, which in turn reinforces PGC-1α-mediated transcription [102]. NSUN7 expression is enriched in the testis and brain. Moreover, mutations in NSUN7 are associated with male infertility [ 103, 104], suggesting that NSUN7 may play distinctive roles that remain unknown.

DNMT2 (or Trdmt1) is currently the sole m ^5^C writer not affiliated with the NSUN family. Initially, categorized as a cytosine DNA methyltransferase, DNMT2 also targets C38 in tRNA ^Asp^. m ^5^C38 in tRNA ^Asp^ by DNMT2 plays a pivotal role in enhancing both translation efficiency and fidelity [200]. Furthermore, DNMT2-mediated tRNA methylation is believed to impact the generation and epitranscriptomic characteristics of tRNA-derived small RNAs (tsRNAs), potentially serving as carriers of paternal epigenetic information [105]. In *Drosophila*, DNMT2 has also been implicated in the methylation of viral RNA, functioning as a defense mechanism against viruses [106]. Recent research conducted in HEK293 cells has expanded the scope of DNMT2 activity to include mRNA methylation, indicating its involvement in this process, which in turn leads to the inhibition of cell proliferation and migration [201].

Overall, many m ^5^C writers are involved in modifying multiple types of RNA substrates, and recent research has not identified a specific m ^5^C writer dedicated to mRNAs. This intricate relationship between m ^5^C writers and their specific targets presents unique challenges and opportunities in isolating and studying the specific functions of individual RNA substrates.

#### Reader

m ^5^C is generally believed to promote RNA stability. A few readers of m ^5^C have been identified. Aly/REF export factor (ALYREF), a known subunit of the mRNA export complex TREX, was first identified as the reader of NSUN2-mediated m ^5^C on mRNA. A large portion of ALYREF’s target mRNAs are m ^5^C labelled. Recognizing m ^5^C via a key residue, K171, ALYREF facilitates the nuclear-cytoplasmic shuttling of m ^5^C-labelled mRNAs. NSUN2 depletion can lead to increased retention of m ^5^C-labelled mRNA as well as the ALYREF protein in the nuclear compartment [109].

Unlike ALYREF, another m ^5^C reader, YBX1, is a cytoplasmic reader that recognizes m ^5^C through the indole ring of W65 in its cold-shock domain (CSD) [202]. By recruiting mRNA stabilizers such as ELAV-like RNA-binding protein 1 (ELAVL1) in cancer and poly(A)-binding protein cytoplasmic 1a (Pabpc1a) in zebrafish embryogenesis, YBX1 facilitates the stability of m ^5^C-labelled transcripts [110]. Intriguingly, YBX1 was also suggested as a translational repressor in zebrafish oocytes and early embryos, as increased translation levels and the unfolded protein response (UPR) were detected in Ybx1-depleted embryos [111]. However, whether this translational repressive function of YBX1 is m ^5^C-dependent remains unknown. Moreover, tsRNAs have the ability to displace the typical mRNA targets of YBX1 under hypoxic stress, which subsequently reduces the stability of the mRNA targets. Nevertheless, whether this displacement is related to m ^5^C remains unexplored [203]. A recent study reported that DNMT2-mediated m ^5^C on mRNA may also serve as a DNA damage code. *In vitro* assays have shown that RAD52 has increased affinity for DNA:RNA hybrids with m ^5^C modifications, suggesting a possible role for RAD52 as an m ^5^C reader that aids in DNA repair processes [204].

YTHDF2, which is widely recognized as an m ^6^A reader, also binds to m ^5^C-modified rRNA and contributes to pre-rRNA processing [112]. Therefore, the severe consequences of YTHDF2 depletion may be attributable to both m ^6^A and m ^5^C modifications.

#### Eraser

Members of the ALKBH family are known dioxygenases that demethylate alkylated DNA and RNA nucleotides [205]. As mentioned above, ALKBH1 has been shown to form f ^5^C at the wobble position (f ^5^C34) of the anticodon on mt-tRNA ^Met^ [194] and cytoplasmic tRNA ^Leu^ via oxidization of m ^5^C34, which expands codon recognition and is essential for mitochondrial translation [108]. Like 5mC on DNA (5mC), m ^5^C can also be oxidized by ten–eleven translocation (TET) family proteins into f ^5^C to 5-carboxycytosine (ca ^5^C). Overexpressing TETs notably elevated the RNA hm ^5^C levels in HEK293T cells [206]. Furthermore, TET2-mediated erasure of m ^5^C is linked to RNA degradation of transcriptionally active ERVs, suggesting that the active erasure of m ^5^C is a posttranscriptional regulatory mechanism [107].

### Pseudouridine (Ψ)

#### Writer

Pseudouridine (Ψ) is a *C* ^5^-glycoside isomer of uridine in which *C* ^5^, instead of *N* ^1^, an atom of the heterocyclic ring, is bonded to the *C* ^1^′ atom of the pentose. As the first RNA modification discovered [ 3, 4] and probably also the most frequent modification other than m ^6^A, Ψ accounts for approximately 0.2%–0.6% of all uridines [207] and is detected in nearly all types of RNA [5]. A family of 13 members, termed the pseudouridine synthase (PUS) protein family, has been identified as Ψ writers. The PUS family can be further divided into two categories. The first includes 12 members that function on their own and directly recognize specific RNA sequences and/or structural features, whereas the other has only one synthase, DKC1, which functions as the catalytic subunit of a small nucleolar RNA-protein (snoRNP) complex and is directed by the H/ACA snoRNA toward its targeted transcripts. Unlike writers of m ^6^A or m ^5^C, which often preferentially target one specific type of RNA, each member of the PUS protein family deposits a Ψ on several RNA species. TRUB1 and DKC1 are believed to mediate the majority of Ψ modifications. Ψ has been widely detected in introns and pre-mRNAs, suggesting that the addition of Ψ may occur quite early in the RNA life cycle [153]. Many PUS proteins, such as PUS1 and PUS7, target mRNAs and tRNAs [208]. Some of these proteins, such as TRUB2 and RPUSD3 [163], also target mt-mRNAs and mt-tRNAs via their mitochondrial localization. Other ncRNAs, such as DKC1, RPUSD4 and PUS10, have additional catalytic activities toward rRNA, mt-rRNA [163], miRNA [209] and other ncRNAs [ 154 , 210].

#### Molecular functions of Ψ

To date, no endogenous erasers or specific readers have been identified for Ψ. Therefore, unlike m ^6^A and m ^5^C, whether Ψ is reversible remains unknown. In addition, the molecular and cellular functions of Ψ have been mostly deduced from the chemical differences between U and Ψ, along with various KO or KD experiments of PUS proteins. The chemical variations between U and Ψ have been shown to alter RNA structure and protein–RNA interactions. The depletion of Ψ writers, which leads to significant loss of Ψ, has been linked to effects on pre-mRNA processing, translation, and mRNA stability.

#### Impact of Ψ on RNA structure

The impact of Ψ on RNA structure is suggested to be largely context dependent [211]. Studies on RNA duplexes, which occur in siRNAs [212] and oligoribonucleotides [213], often find that Ψ is a stabilizing modification [214]. Unlike U, which adopts a *C*-2′-endo sugar conformation, Ψ preferentially adopts a *C* ^3^′-endo sugar conformation because of hydrogen bonding interactions with its phosphate backbone. This modified conformation enhances the stability of the RNA backbone and base, contributing to the stabilization of RNA duplexes [212]. The presence of Ψ can also increase the rigidity of the backbone of the polypyrimidine tract of adenovirus pre-mRNA, resulting in splicing abnormalities [215]. On the other hand, Ψs were observed to have slight destabilizing effects when positioned in single-stranded loop regions [216] .

Current studies on the effects of Ψ on eukaryotic mRNA structure are limited. Chemical probing analyses of both unmodified and modified mRNAs within various cellular contexts may constitute an initial approach that may reveal the impact of Ψ on the structure of mRNAs and their potential effects on mRNA metabolism.

#### Impacts of Ψ on RNA-protein interactions

Ψ can stabilize, destabilize, and alter RNA structural equilibria *in vitro*, which could in turn impact RNA-protein interactions and mRNA processing. Unlike modifications such as m ^6^A, which contains an additional methyl group that can be specifically accommodated by its readers, Ψ is formed by the isomerization of U and therefore may not have dedicated readers. Instead, Ψ may change the binding affinities of RBPs for their canonical targets. Ψ-containing RNAs reduce the binding of certain proteins, potentially due to altered RNA structure. As mentioned above, the increased rigidity of the backbone of the polypyrimidine tract, caused by Ψ at two sites, inhibits the binding of the splicing factor U2AF and causes splicing defects [215]. Similarly, Ψ also reduces the affinity of the Sm protein to U7 snRNA [217], of the splicing factor MBNL1 to expand repeats [218], and of PUM2 to RNAs harboring its binding motif *in vitro* [219]. In addition, synthetic mRNAs modified with Ψ, including mammalian RNase L *in vivo* [220] and *Escherichia coli* RNase E *in vitro* [221], exhibit reduced cleavage by RNases.

On the other hand, Ψ-modified RNAs also attract some proteins. The yeast methionine aminoacyl-tRNA ^Met^ synthetase MetRS showed increased affinity for Ψ-modified RNA *in vitro*. Mutation of Ψ to C in an endogenous mRNA also reduced MetRS binding, further confirming that MetRS functions as a reader of Ψ-containing mRNAs [222]. The yeast RNA helicase Prp5 bound more to U2 snRNAs with Ψ42 and Ψ44, potentially because of Ψ-induced structural changes in the branch-site recognition region [223]. Ψ has also been shown to reduce foreign RNA sensing and innate immune responses potentially by increasing the affinity between Ψ-modified RNA and the cytosolic innate immune receptor RIG-I, which prevents the filament formation of RIG-I downstream [ 224 , 225].

Although most of these studies are *in vitro* and none has investigated the effects of endogenous Ψ in mammalian systems, they do indicate that Ψ has the ability to alter RNA structure and RBP binding. Therefore, the identities of the endogenous readers of Ψ, especially in mammalian cells, remain an outstanding question.

#### Impact of Ψ on mRNA metabolism

The alterations in RNA structure, snRNA-pre-mRNA pairing and RBP binding induced by Ψ are expected to impact mRNA metabolism. This notion is further supported by investigating the distribution of endogenous Ψs and the consequences of the depletion of Ψs. A recent study revealed that Ψ is enriched near splice sites in pre-mRNAs in the human hepatocellular carcinoma cell line HepG2 and that depletion of Ψ writers, specifically PUS1, PUS7 and RPUSD4, leads to changes in alternative pre-mRNA splicing and 3′ end processing [153].

Ψ also impacts translation efficiency and fidelity by mediating codon interpretation, although different studies have some discrepancies regarding the direction of its impact. For example, when Ψ is introduced into a specific phenylalanine codon (UUU), it slows peptide synthesis [226] and reduces the yield of full-length peptides *in vitro* [ 227, 228], in contrast with the enhanced translation observed with fully substituted therapeutic mRNAs in live cells [229]. This inconsistency is possibly due to additional translational controls *in vivo* that are not present in simpler *in vitro* systems. Additionally, Ψ, often at the first and third positions of a codon, can cause amino acid misincorporation [230]. This misincorporation, such as replacing phenylalanine by leucine or valine, varies depending on the Ψ position and the specific tRNA involved and occasionally affects translation efficiency [226]. Furthermore, Ψ modifications at stop codons can promote readthrough, allowing the ribosome to bypass typical stop codons and extend protein products. Evidence supporting this notion have been reported in yeast [ 231 , 232], mammalian cell lines [ 233, 234] and tissues [157], albeit with varying stoichiometries of Ψ and readthrough efficiencies, suggesting a context-dependent regulatory mechanism of this Ψ-mediated stop codon readthrough.

Consistent with the stabilizing effect of Ψ in RNA duplexes, the presence of Ψ in mRNAs is also suggested to promote mRNA stability. mRNA abundance decreases following the depletion of Ψ writers in yeast and mammalian cells [235], and the induction of Ψ at specific sites increases the mRNA lifespan [157]. Interestingly, the mRNA targets of a pseudouridine synthase (TgPUS1) in *Toxoplasma gondii*, a protozoan parasite, had a longer half-life and were more abundant in TgPUS1-mutant parasites [236], suggesting that the regulatory role of Ψ in RNA stability may again be context-dependent. Overall, while the impact of Ψ on mRNA metabolism has been extensively studied, further research will be necessary to uncover detailed mechanistic insights into this context-specific regulation of Ψ.

### *N* ^4^-acetylcytidine (ac ^4^C)

*N* ^4^-acetylcytidine (ac ^4^C), the sole acetylation event identified in eukaryotic RNA, was first reported in yeast [145] and *Escherichia coli* tRNA [237] and was later detected in human and yeast 18S rRNA [ 146, 147 ]. An emerging body of recent evidence supports the presence of ac ^4^C on mRNAs as well [ 148– 150 ]. The ac ^4^C on mRNA has dual effects. When in a coding sequence (CDS), ac ^4^C strongly enhances translation elongation by promoting interactions with cognate tRNAs, whereas ac ^4^C in the 5′UTR inhibits translation initiation by generating 5′UTR repressive structures or disrupting interactions with tRNA _i_^Met^ [149]

The measurement of ac ^4^C by MS-based or RIP-seq-based methods initially varied across studies [ 148, 238 ]. Even with recently established single nucleotide-resolution methods such as ac ^4^C-seq and RedaC:T-seq, the number and distribution of candidate ac ^4^C sites, especially those on mRNAs, differ across studies [ 149 , 150]. Nevertheless, it is widely accepted that ac ^4^C on RNA is catalyzed by a single writer, N-acetyltransferase 10 (NAT10). NAT10 transfers an acetyl group from acetyl-CoA to the exocyclic N4-amine of cytidine. Three domains of NAT10, the acetyltransferase, ATPase and RNA-binding domains, are essential for this acetylation event in eukaryotic RNA [ 147, 239]. NAT10-mediated acetylation also requires adaptors for target specificity. NAT10 interacts with snoRNA (SNORD13) to mediate the acetylation of 18S rRNA in human cells and interacts with the protein adaptor THUMPD1 to mediate the acetylation of tRNA ^Leu^ and tRNA ^Ser^ [ 146, 240 , 241]. Although depletion of these two adaptors leads to considerable loss of ac ^4^C, they generally do not impact cell viability or proliferation [ 240, 242]. In contrast, depletion of NAT10 is detrimental, leading to defects in pluripotency, cell cycle regulation and cell migration, which suggests that acetylation of other substrates of NAT10, perhaps mRNAs, may be essential for those biological events [ 148, 151, 152, 242 , 243]. However, NAT10 is also involved in regulating microtubule and histone acetylation [ 244– 246], suggesting that the consequences of NAT10 deletion cannot be attributed solely to the potential functions of the ac ^4^C mRNA. Therefore, the exact effects of ac ^4^C on mRNAs remain unexplored. Uncovering how NAT10 targets mRNAs is another major challenge. Several factors, such as the subcellular localization of NAT10 [ 247, 248] and potential adapters for mRNAs, may contribute to this degree of mRNA acetylation. Dissecting the target selection mechanisms of NAT10 will facilitate further understanding of the function of ac ^4^C and may also provide some explanation for the large discrepancies across different studies.

### *N* ^1^-methyladenosine (m ^1^A)

*N* ^1^-methyladenosine (m ^1^A), whose level is approximately 10-fold lower than that of m ^6^A, remains a key modification in tRNAs and rRNAs and affects their processing, secondary structure, and stability. For example, in mt-tRNA ^Lys^, the methyl group of m ^1^A9 disrupts base pairing, inducing the cloverleaf structure and the correct formation of the DHU loop [249]; m ^1^A58 in the initiator methionine tRNA (tRNA ^iMet^) of yeast and mammals promotes a unique A54-A58 interaction and stabilizes its structure [250]; and m ^1^A947 in the mitochondrial 16S rRNA (mt-16S rRNA) may also facilitate the stabilization of the mitochondrial ribosome. By ensuring proper processing and stabilizing of tRNAs and rRNAs, m ^1^A on those RNAs generally promotes translation efficiency.

Recent studies have confirmed the presence of m ^1^A in eukaryotic mRNAs. Hundreds of candidate m ^1^A sites were detected, albeit with very low stoichiometries [ 114, 251 – 253]. These m ^1^A sites are suggested to have dual functions in translation, depending on their location. In CDS regions of mitochondrial and nuclear mRNAs, m ^1^A often leads to translational repression, potentially because of its disruptive impact on base pairing and, consequently, ribosomal scanning, whereas m ^1^A in the 5′UTR is associated with increased translation, as its positive charge may destabilize the secondary structure in the 5′UTR, which in turn promotes translation initiation. An interesting exception of a highly methylated site is detected in the mitochondrial-encoded ND5 gene, the function of which remains unknown.

#### Writer

Writers of m ^1^A mainly belong to the tRNA methyltransferase (TRMT) family. TRMT61A and TRMT6 form a heterotetramer methyltransferase complex that installs m ^1^A in tRNA and some sites of mRNAs in the cytoplasm. In this complex, TRMT61A, which contains an S-adenosyl-L-methionine (SAM) binding pocket, is the catalytic subunit, whereas TRMT6 mediates RNA binding. The TRMT6/61A complex specifically targets A58 in tRNA, which is located within the T-loop. Additionally, TRMT6/61A-mediated m ^1^A sites in mRNAs or lncRNAs are enriched in GC-rich regions and tRNA-like motifs, suggesting that the target recognition of TRMT6/61A is structure-dependent [ 114, 115]. In the mitochondria, TRMT61B mediates m ^1^A modification on mt-16S rRNA and possibly mt-tRNA, with a preference for a consensus YMRAW motif [ 114, 116]. TRMT10C, in complex with SDR5C1, forms m ^1^A9 on mt-tRNA [117]. TRMT10B also has tRNA ^Asp^-specific methyltransferase activity *in vitro* [254]. An additional writer that does not belong to the TRMT family, NML (also known as RRP8), is found in the nucleus and mediates the m ^1^A modification of 28S rRNA [113] .

#### Reader

The YTH domain is likely a specialized structure for recognizing the methyl group of RNA modification, as the four YTH family proteins (YTHDF1, YTHDF2, YTHDF3, and YTHDC1), which were initially identified as m ^6^A readers, also show binding affinity with m ^1^A, albeit at a lower level [255]. Recent studies suggested that m ^1^A promotes mRNA destabilization via YTHDF2 and YTHDF3 [ 122, 123]. Intriguingly, a recent study also revealed that increased m ^1^A levels contribute to the development of pathological status via a novel reader, TDP-43. TDP-43 preferentially binds to m ^1^A-containing CAG repeat RNA, which induces the sequestration of TDP-43 into stress granules and consequently its cytoplasmic mislocalization, as observed in neurological diseases [124].

#### Eraser

Like m ^6^A and m ^5^C, the erasure of m ^1^A is mainly mediated by ALKBH family demethylases, specifically ALKBH1 and ALKBH3 in the cytosol and ALKBH7 in the mitochondria. ALKBH1 preferentially targets m ^1^A58 in tRNA, whereas ALKBH3 facilitates demethylation of both tRNA [ 118, 119] and mRNA [120]. The mitochondrion-localized ALKBH7 demethylates both m ^1^A and m _2_^2^G on mt-pre-tRNA and mt-dsRNA, modulating mitochondrial polycistronic RNA processing [121]. m ^1^A in tRNA can also be erased by the m ^6^A demethylase FTO. The demethylation of m ^1^A on tRNA by FTO was shown to negatively affect translation efficiency in both *in vitro* and *in vivo* reporter assays [50] .

### *N* ^7^-methylguanosine (m ^7^G)

*N* ^7^-methylguanosine (m ^7^G), the methylation site of guanine at position *N* ^7^ in RNA, was initially identified at the 5′ cap (m ^7^GPP) of mRNA. m ^7^GPP enhances the stability of transcripts and facilitates various 5′ cap-related biological processes, such as pre-mRNA processing and nuclear export [256]. The presence of m ^7^G was later detected at internal positions within mRNAs, tRNAs, and rRNAs. Among these sites, the methylation of guanine at position 46 (m ^7^G46) within the variable loop region of tRNA is considered the most prevalent m ^7^G-modified site [ 257 , 258].

#### Writer

Most m ^7^G methyltransferases are members of the SAM-dependent methyltransferase family. The METTL1/WDR4 complex is the most well-characterized m ^7^G writer, of which the substrates include tRNAs, mRNAs, and miRNAs [ 125, 126]. Cryo-EM revealed that the disordered N-terminal of METTL1, when bound with SAM or SAH, coordinates and stabilizes the assembly of tRNA, the catalytic loop of METTL1 and the C-terminal helix of WDR4 near the catalytic pocket of METTL1. The phosphorylation of Ser27 in the N-terminal of METTL1 inhibits proper organization of the catalytic pocket, providing a structural explanation for the posttranslational regulation of m ^7^G modification. WDR4 mainly acts as a scaffold for METTL1 and the tRNA T-arm. When acting on mRNAs, the METTL1–WDR4 complex has also been suggested to recognize regions with tRNA-like structures [259]. A complex of RNMT and RNMT-activated small protein (RAM) installs m ^7^G for the 5′cap structure [ 128– 130]. This complex is activated by CDK1-cyclinB1-mediated phosphorylation. CDK1-cyclinB1 also blocks the binding of inhibitory proteins, such as nuclear protein subunit α2 (KPNA2), to the RNMT-RAM complex [260]. Some types of RNAs, including snRNAs, snoRNAs [ 131, 132], tRNAs [261], telomerase RNAs [ 262 , 263] and some sncRNAs [143], contain a hypermethylated cap (m ^2,2,7^G-cap or TMG-cap), which is installed by TGS1. WBSCR22, with TRMT112 as a cofactor, mediates the methylation of G1639 in human 18S rRNA [264] .

#### Reader

Four readers have been reported to recognize the m ^7^G cap and affect RNA maturation, nuclear export, and translation. The nuclear cap binding protein complex (CBC), which consists mainly of the cap-binding protein NCBP1 (CBP80) and the adaptor NCBP2 (CBP20), recognizes the m ^7^G cap and facilitates the maturation [133] and export of mRNA [134]. CBCs are also detected in the cytoplasm and participate in the translation of viral RNA [265]. eIF4E, a eukaryotic translation initiation factor, binds to the m ^7^G cap and promotes the translation of a group of transcripts. Structural data revealed that the positive m ^7^G cap stacks between W102 and W56, interacts with the aromatic residues of eIF4E, and forms a cation–π sandwich [266]. Nuclear eIF4E also acts in the processing and export of a selection of mRNAs [ 135 – 137]. The import of eIF4E into the nucleus is mediated via direct interaction between importin 8 and the cap-binding domain of eIF4E; therefore, only cap-free eIF4E is imported [267]. Recent studies have revealed that eIF4E can form a complex with RNMT, which promotes the capture of newly capped RNA by eIF4E [268]. In addition to eIF4E, another initiation factor, eIF3d, also has cap-binding ability and mediates translation initiation [269]. Poly(A)-specific ribonuclease (PARN), a deadenylase, binds to both the poly(A) tail and the m ^7^G cap. A tryptophan residue in the RRM domain of PARN forms a stacking interaction with m ^7^G, and the R3H domain is suggested to stabilize the cap-binding pocket [ 270 , 271]. This cap-binding activity of PARN also promotes its deadenylation activity [ 138 , 139]. Additional readers have also been suggested to bind to the TMG cap on ncRNAs and mediate their localization, including snuportin-1 (SNUPN), which transports snRNAs from the cytoplasm to the nucleus [ 140, 141], and Exportin-1 (XPO1/CRM1), which transports U3 snoRNAs from Cajal bodies to the nucleoli [142], and pri-miRNAs to the cytoplasm [143].

By applying RNA affinity purification followed by mass spectrometry, a recent study identified QKI as a specific reader for internal m ^7^G on mRNA. Over one thousand QKI-bound, m ^7^G-modified mRNAs were identified and enriched for a consensus GANGAN motif. One of the QKI isoforms, QKI7, interacts with G3BP1, a core component of the stress granule (SG), and mediates the transport of m ^7^G-modified mRNAs into stress granules under stress conditions, which reduces their translation [144].

#### Molecular functions of m ^7^G

m ^7^G, with its positive charge, may also have an effect on base pairing, RNA structure and RNA-protein interactions, eliciting regulatory effects without a specific reader. For example, m ^7^G46, one of the most prevalent tRNA modifications, forms a C13-G22-m ^7^G46 base triple interaction that maintains tRNA structural integrity [272]. In yeast, mutation of m ^7^G leads to the loss of m ^7^G46 and subsequent degradation via the rapid tRNA decay (RTD) pathway [273]. The m ^7^G46 modification of tRNA may also increase the decoding ability of the tRNA codon and therefore prevent ribosome pausing and collisions [ 274, 275]. Moreover, m ^7^G11 on *let-7* pri-miRNA promotes its processing via the inhibition of local G-quadruplex structures and, in turn, promotes migration in the human lung cancer cell line A549 [276].

Two independent groups have reported the internal m ^7^G of mRNAs, both of which suggest a translation-promoting function of those internal m ^7^Gs [ 259, 277]. Dynamic regulation of internal m ^7^G was also observed under stress conditions, suggesting its potential functions in the stress response [ 144, 277]. The internal m ^7^G in mRNAs may cause local structural changes that affect RNA-protein interactions or be directly recognized by specific reader proteins, which downstream modulate RNA metabolism. Currently, only one such reader of internal m ^7^G has been identified [144]. Additionally, no specific demethylase has been identified to target m ^7^G, and our understanding of m ^7^G regulation and function is incomplete at present.

## Physiological Roles of RNA Modifications in Reproduction and Development

**Figure 2:**
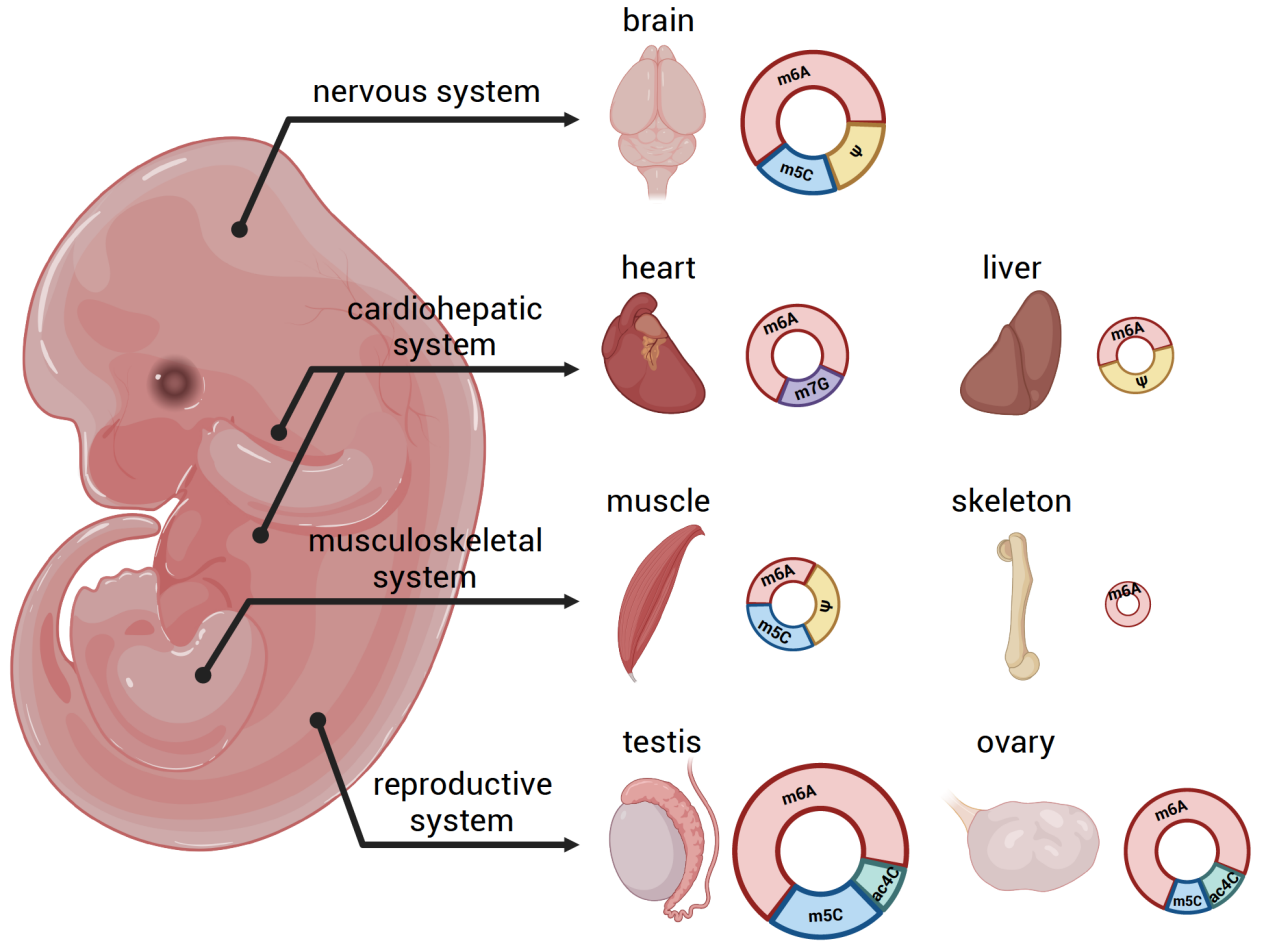
Figure 2 RNA modifications in mammalian development Recent studies uncovered crucial roles of RNA modifications and their modifiers in mammalian development, especially in nervous system, cardiohepatic system, musculoskeletal system and reproductive system. The size and space of circles corresponds to the number of RNA modifiers identified to be functional in specific system.

RNA modifications represent a sophisticated layer of co- and post-transcriptional regulation that underscores the complexity of developmental biology. Recent studies, with advanced *in vitro* culture systems and low-input detection methods, have revealed several unique roles of RNA modifications and their modifiers during mammalian development, especially in gametogenesis ( Figure 2). In this section, we summarize recent findings that highlight the regulatory functions of epitranscriptomics in mammalian development and related diseases.

Recent studies uncovered crucial roles of RNA modifications and their modifiers in mammalian development, especially in nervous system, cardiohepatic system, musculoskeletal system and reproductive system. The size and space of circles corresponds to the number of RNA modifiers identified to be functional in specific system.

### Spermatogenesis

**Figure 3:**
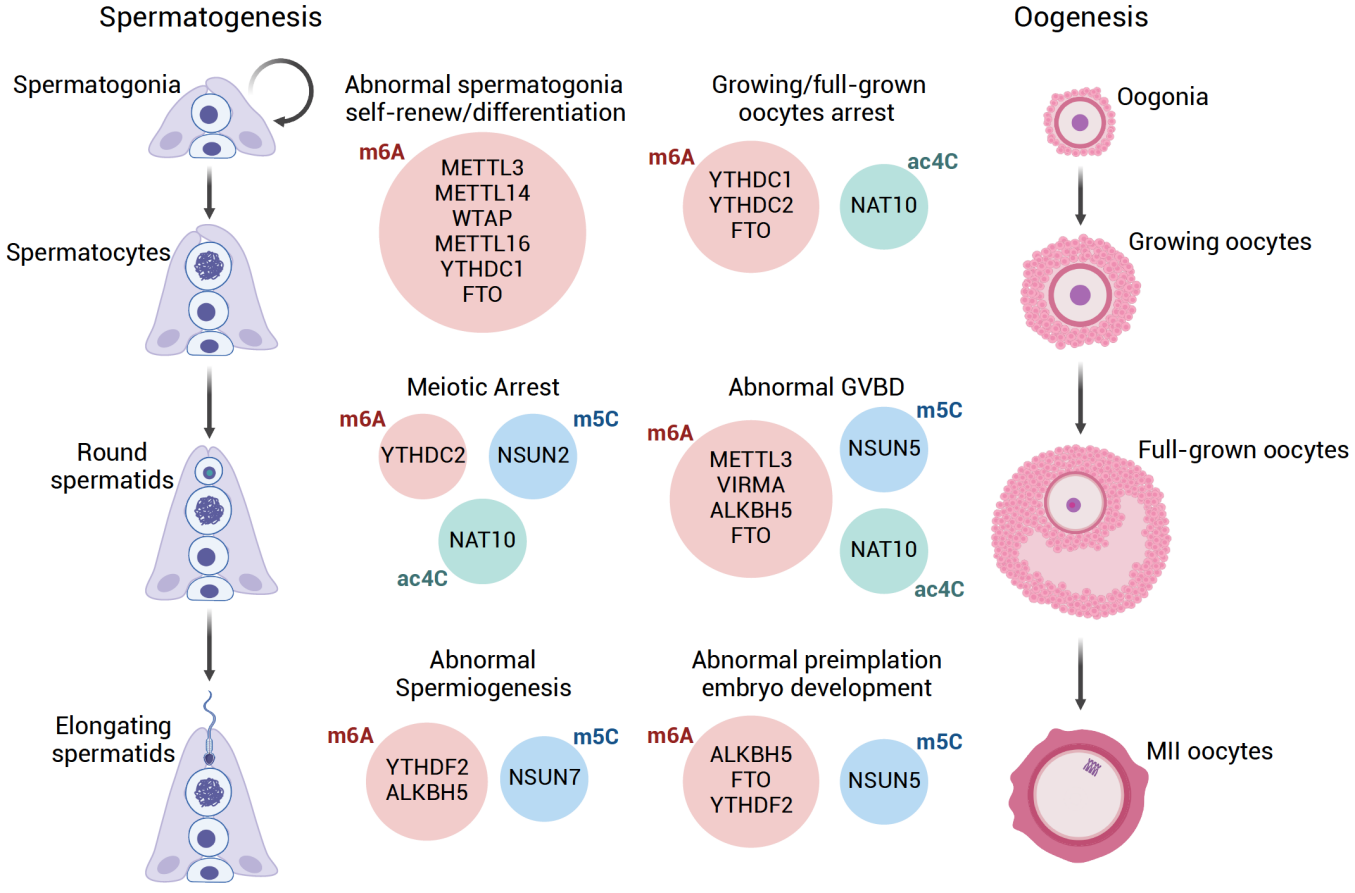
Figure 3 Roles of RNA modifiers in the reproductive system Gametogenesis, including spermatogenesis and oogenesis, is one of the most sophisticated developmental process and has been reported to have the largest number of RNA modifiers to be involved in. Different types of RNA modifications or even different modifiers of same RNA modification result in gametogenesis arrested in different stages.

Mammalian spermatogenesis is an intricate process in which a diploid spermatogonial stem cell (SSC) undergoes a series of steps-differentiation, mitotic expansion, meiotic division, and spermiogenesis-ultimately transforming into haploid, motile spermatozoa. Each stage of this process is highly specialized and requires precise spatiotemporal control of gene expression. The epitranscriptome contributes a significant mechanism that supports such stringent control ( Figure 3).

#### m ^6^A

As mentioned above, one mammalian m ^6^A demethylase, ALKBH5, was first identified in the context of spermatogenesis in 2013. ALKBH5, which promotes mRNA processing and export by removing m ^6^A, is a critical regulator of spermatogenesis but does not play a key role in other tissues. *Alkbh5*-deficient male mice generally exhibit a normal phenotype, except for impaired fertility, with spermatocytes arrested at metaphase [186]. Later, two groups independently outlined a comprehensive m ^6^A landscape in spermatogenesis via immunoprecipitation (IP)-based methods. When the m ^6^A writers *Mettl3* or *Mettl14* are depleted via *Vasa*-Cre, which is activated in embryonic germ cells, loss of m ^6^A was observed, as were defects in SSCs due to dysregulation of splicing, translation and potentially stability [ 11, 12]. Intriguingly, in advanced germ cells ( *i*. *e*., post-SSC differentiation), ablation of both METTL3 and METTL14, but not either, affects the translation of key spermiogenesis genes, suggesting that these two enzymes may be partially redundant in spermiogenesis [12]. Another m ^6^A writer, METTL16 (the orthologue of *Caenorhabditis elegans* METT-10), is also essential for male fertility, as conditional depletion of METTL16 in the germline results in early spermatogenetic arrest in mice. In *Caenorhabditis elegans*, when exposed to a nutrient-rich diet, METT-10 installs m ^6^A on the 3′ splice site of a specific transcript, *sams*, which blocks the binding of the essential splicing factor U2AF35 and leads to defective splicing. Blockade of U2AF35 by m ^6^A was also observed in the HeLa cell line via a transgene reporter assay, suggesting that this mechanism is likely conserved in mammals and may be responsible for male infertility in METTL16-deficient mice [44].

In addition to writers, multiple readers of m ^6^A are also vital players in spermatogenesis. YTHDF2, which is most abundant in spermatocytes, facilitates the clearance of spermatogonia-specific transcripts in pachytene spermatocytes and ensures proper spermatogenic progression [66]. YTHDC1, which is highly expressed in germlines from embryonic germ cell stages, is essential for the survival of spermatogonia, as the absence of YTHDC1 in male germ cells leads to the Sertoli-cell-only phenotype [59]. YTHDC2 functions as an RNA helicase ensuring meiotic entry and progression in gametogenesis. Despite being an m ^6^A reader, a recent study suggested that the function of YTHDC2 in spermatogenesis is not m ^6^A-dependent and is therefore worthy of further exploration [13]. PRRC2A, which interacts with mRNA metabolic factors such as YBX proteins, mediates the downregulation of spermatogonia-specific genes as well as the translation of meiosis-related genes during meiosis prophase. PRRC2A-deficient spermatocytes present multiple meiotic defects, including XY asynapsis, and are arrested at metaphase I. This bidirectional regulation of PRRC2A is different from its stabilizing role in oligodendroglial specification, suggesting a potentially germ cell-specific function [76].

The reversibility of m ^6^A, *i*. *e*., proper demethylation of m ^6^A, is equally critical for expression regulation. Not surprisingly, erasers of m ^6^A, ALKBH5 and FTO, are also suggested to be essential in spermatogenesis. ALKBH5-mediated demethylation regulates the splicing and specific degradation of transcripts with longer 3′UTRs during the meiosis-to-spermiogenesis transition [54]. FTO-mediated demethylation regulates the translation of the androgen receptor (AR) and promotes Leydig cell maturation. Moreover, although the direct targets of FTO in male gem cells remain unknown, the proliferation of undifferentiated spermatogonia is impaired in *FTO*-KO mice in an age-dependent manner [51]. Notably, *FTO*-KO mice also exhibit an obesity phenotype [278]. Obesity-related metabolic and hormonal dysregulation may also contribute to age-dependent defects in spermatogenesis. Therefore, the direct, mechanistic functions of FTO in the testis remain to be elucidated.

#### Other RNA modifications

In addition to the dominant m ^6^A, m ^5^C and ac ^4^C have been shown to be important in spermatogenesis. Depletion of the m ^5^C writer *Nsun2* results in spermatogenic arrest at the leptotene and zygotene (LZ) stages. NSUN2, which is localized to the chromatoid body in round spermatids, may also have unique functions during spermiogenesis [86]. The depletion of *Nsun7* in mice does not cause arrest at any specific stage of spermatogenesis. However, these mice are infertile, as mutant sperm largely lose flexibility in the flagellar midpiece and, subsequently, progressive motility [104]. Another intriguing case is the deletion of DNMT2, which does not affect male fertility but changes the profile of small RNAs carried in mature sperm. These small RNAs in sperm are suggested to convey paternal experiences, such as a high-fat diet, to offspring. Therefore, the germline presents a unique case in which RNA modifications are pivotal not only in controlling the expression of genes important for development but also in composing the epigenetic information passed to the next generation [105].

Using HPLC-MS/MS, ac ^4^C on total RNA and mRNA has been quantified in multiple mouse tissues. Compared with other somatic tissues, the testis and epididymis have relatively high levels of ac ^4^C, and the abundance of ac ^4^C during spermatogenesis is dynamic, gradually decreasing from LZ spermatocytes to round spermatids. Depletion of Nat10 in male germ cells led to a significant reduction in ac ^4^C in testicular mRNA, as expected but also a large increase in m ^6^A, suggesting potential crosstalk between RNA modifications. Spermatocytes lacking NAT10 cease developing around the pachytene stage, even though transcriptional anomalies can be detected as early as the spermatogonial phase. Nevertheless, as discussed above, given the multifaceted nature of NAT10, the implications of its deletion cannot be straightforwardly interpreted as the potential functions of ac ^4^C on mRNA. The dysregulated transcriptome in NAT10-deficient male germ cells may be a cumulative result of multiple disrupted biological processes [151].

Overall, although multiple writers have been shown to be essential for spermatogenesis, our understanding of the dynamics and roles of m ^5^C and ac ^4^C, as well as other RNA modifications such as Ψ, remains significantly incomplete.

## Oogenesis and Preimplantation Embryonic Development

Oogenesis, the development of female gametes, or eggs, is differentially regulated but equally intricate compared with spermatogenesis. The female germline enters meiosis around embryonic days E13.5 to E15.5. The immature oocytes are arrested in the diplotene stage of meiosis prophase I after birth. The oocytes are subsequently surrounded by somatic granulosa cells, which form primordial follicles ( *i* . *e*., oocytes pause at the diplotene stage in meiotic prophase I). These follicles remain quiescent until the onset of sexual maturity. Upon hormonal signaling, the oocytes increase in size and accumulate extra layers of granulosa cells, transitioning into primary, secondary, and pre-antral/antral follicles. During this period, the oocytes also resume meiosis, which is characterized by germinal vesicle (GV) breakdown, chromosome condensation and polar body extrusion.

A key difference between oogenesis and spermatogenesis is that while male germ cells become motile sperm with minimal cytoplasm, female germ cells build an extremely complex cytoplasm with a substantial amount of RNAs as resources for early embryonic, especially zygotic, development. These maternal RNAs only initiate translation after fertilization. The storage, translational transition and later degradation of maternal RNAs may rely heavily on RNA modifications and their readers. The dysregulation of RNA modifications in oogenesis may not only disrupt the generation of mature oocytes but also have an adverse effect on fertilization and preimplantation embryonic development ( Figure 3 ).

### m ^6^A

Recent studies have revealed that m ^6^A modification is a key means of controlling gene expression during oocyte maturation and the maternal-to-zygotic transition. A large portion of maternal RNAs stored in mature oocytes are m ^6^A-modified, and m ^6^A modification is highly dynamic during early embryonic development [ 7, 279 ]. A most recent study using single-cell m ^6^A sequencing (scm ^6^A-seq) further reported m ^6^A-dependent asymmetries in the blastomeres of two-cell embryos [280], which may contribute to the unequal developmental potential of the two blastomeres of two-cell embryos [ 281– 283]. Additionally, multiple writers, readers and erasers of m ^6^A are essential and non-redundant in oocyte and early embryonic development.

The m ^6^A writer METTL3 is essential for oogenesis, potentially from the primordial germ cell (PGC) stage. When *Vasa*-Cre, which is activated in late PGCs, is used, the *Mettl3*-deficient ovary is morphologically abnormal, with no clear follicle observed [26]. When *Gdf9*-Cre or *Zp3*-Cre, which are activated in the primordial follicle stage, is used, *Mettl3*-deficient oocytes fail to undergo germinal vesicle breakdown (GVBD) ( *i*. *e*., cannot resume meiosis), and few follicles beyond the primary stage are observed [ 25, 26]. DNA damage accumulation [25] and aberrant RNA clearance [280] were also observed in those oocytes. METTL3 may also target a few meiosis-associated and DNA repair-associated transcripts, such as *Itsn2*, which enhances their stability and regulates oocyte meiosis [25]. Upon fertilization, these *Mettl3*-deficient oocytes fail to develop past the two-cell stage [ 8, 26]. During zygotic genome activation (ZGA) in two-cell embryos, m ^6^A newly deposited via METTL3 also regulates mRNA decay beyond the two-cell stage [279]. In blastocysts, METTL3 functions as a key factor regulating naïve pluripotency, ensuring timely exit from the naïve state and proper lineage priming. *Mettl3* ^–/–^-KO blastocysts obtained from crossing *Mettl3* ^+/–^ mice and *Mettl3* ^–/–^-KO naïve ESCs exhibit significant loss of m ^6^A in mRNA and fail to exit naïve pluripotency. Eighty percent of naïve pluripotency-promoting genes, such as *Nanog* and *Klf2*, are marked with m ^6^A. Upon *Mettl3* depletion, the expression of naïve pluripotency markers in naïve cells is further amplified, which stabilizes the naïve pluripotency circuitry [284]. Similar to METTL3 deficiency, a lack of KIAA1429, a key cofactor of the METTL3/METTL14 complex, in oocytes leads to failure of GVBD and meiotic resumption. KIAA1429-deficient oocytes exhibit abnormal accumulation of RNAs as well as splicing defects [38]. Intriguingly, a recent study revealed that transcripts of transposable elements, such as MTA, a phylogenetically young retrotransposon of the LTR family (MaLR subfamily), are also m ^6^A-modified and likely targets of the METTL3/METTL14 complex. The abundance of stage-specific retrotransposons, such as MTA and MERVL, is regulated via m ^6^A and is potentially critical for oocyte maturation and early zygotic development [279]. In addition to the METTL3/METTL14 complex, a second m ^6^A writer, METTL16, is indispensable for early embryonic development, as a lack of METTL16 in mouse 16-cell embryos leads to a decrease in the transcription of its target MAT2A, a SAM synthetase, followed by transcriptome-wide disruption at the 64-cell stage and developmental arrest around the time of implantation [45].

Many of the known readers of m ^6^A have been investigated in the context of oogenesis or early embryonic development, and some of them have shown unique functions that cannot be compensated for by other readers. The most well-researched reader is probably YTHDF2, which is required for both oocyte development and early zygotic development. Upon the loss of YTHDF2 from the primordial follicle stage, normal numbers of MII oocytes can be produced. However, these MII oocytes lose the ability to support early zygotic development. Transcriptomic analysis revealed that a group of transcripts modified with m ^6^A and enriched for the YTHDF2-binding consensus were stabilized upon loss of YTHDF2 when they were expected to be degraded during the GV to MII transition, leading to an abnormal transcript dosage in *YTHDF2*-KO MII oocytes [67]. Although YTHDF1, YTHDF2, and YTHDF3 are suggested to have different cellular functions, *Ythdf1*- and *Ythdf3*-deficient mice are viable and fertile. On the basis of the observed Mendelian ratio of offspring during the attempt to generate *Ythdf* triple-KO mice, as well as RNA-seq and eCLIP data in mESCs, it is suggested that deficiency of *Ythdf1* and *Ythdf3*, but not *Ythdf2*, can be mostly compensated by other YTHDF readers [26]. The other two YTH family readers, YTHDC1 and YTHDC2, play critical roles during oocyte maturation. YTHDC1, in association with 3′end processing factors such as SRSF3, regulates polyadenylation in oocytes. Deficiency of *Ythdc1* in oocytes leads to arrest at the primary follicle stage [59]. YTHDC2 potentially plays a similar role in oogenesis and spermatogenesis, as failure to reach the pachytene stage was also observed in the oocytes of *Ythdc2*-KO mice [9]. As mentioned above, retrotransposon transcripts also carry m ^6^A modifications. m ^6^A in retrotransposon transcripts and mRNAs can be recognized by IGF2BP2 and consequently stabilized in oocytes, and depletion of IGF2BP2 results in decreased MTA RNA level in oocytes [ 56 , 279].

Abnormal RNA metabolism in oocytes and early embryos is also observed when erasers of m ^6^A are absent. FTO specifically demethylates RNA transcribed from long interspersed element-1 (LINE1) in LINE and promotes its stability. Lack of FTO also disrupts the local open chromatin state, potentially in a carRNA-dependent manner, which in turn affects the transcription of LINE1-containing genes. FTO-LINE1s are important for oocyte maturation and early development [52]. Deficiency of ALKBH5 during oocyte maturation leads to RNA accumulation, which can be partially rescued by reducing the m ^6^A reader IGF2BP2, suggesting that ALKBH5 is a key counteracting factor of m ^6^A-mediated stabilization in oocytes [56].

### Other RNA modifications

m ^5^C, which extensively occurs in the mRNAs of oocytes and early embryos, also plays a key role in regulating oocyte maturation and the maternal-to-zygotic transition (MZT). The two different types of m ^5^C, which are deposited by NSUN2 and NSUN6, respectively, were first summarized in the context of oogenesis. Maternal depletion of *Nsun2* in Drosophila results in a lack of maternal m ^5^C mRNAs, cell cycle delays and failure of MZT [285]. In zebrafish embryos, YBX1 promotes mRNA stability in an m ^5^C-dependent manner [110]. NSUN5 is highly expressed in mammalian oocytes. In mice, *Nsun5* deletion leads to a significant decrease in the m ^5^C level in ovaries and affects translation efficiency as well as splicing of a few genes, such as *Mad2l2*, *Gdf9* and *Brd8*, which may contribute to the observed defects in ovarian function and embryonic development [94].

Like spermatogenesis, a lack of NAT10, the ac ^4^C writer, in premeiotic oocytes leads to developmental arrest around the pachytene stage. Deletion of NAT10 in primordial follicles leads to a substantial portion of oocytes failing to complete GVBD. The *Nat10*-deposited ac ^4^C modification was detected on mRNAs of CCR4-NOT complex components such as *Cnot6l*, *Cnot7* and *Btg4*, potentially regulating their stability and ensuring proper CCR4-NOT-dependent degradation. In addition, *Nat10*-deposited ac ^4^C modification may also increase the translation efficiency of a group of housekeeping genes. Together, *Nat10*-deposited ac ^4^C modification is essential for mouse oocyte development [152].

## Organogenesis and Developmental Disease

Organogenesis is a complex and highly coordinated process through which stem cells proliferate, differentiate and are organized into functional structures. Disruptions in the intricate processes of organogenesis can directly lead to premature lethality and congenital anomalies, often termed developmental diseases or birth defects. Understanding the mechanisms of organogenesis is crucial for revealing the origins of many developmental diseases and provides the basis for developing preventive and therapeutic strategies. Research continues to shed light on the regulatory mechanisms of organogenesis, including the potential roles of RNA modifications, offering new perspectives for better management and treatment of such conditions in the future.

### Nervous system

#### m ^6^A

Given the complexity of the nervous system, it is not surprising that the epitranscriptome is likely to play a pivotal role during its development. In the developing mouse cerebellum, m ^6^A modifications, modifiers and effectors are spatiotemporally dynamic, suggesting that m ^6^A modifications are precisely controlled and critical for proper neurodevelopment [29].

Multiple studies have focused on the functions of the METTL3/METTL14 complex in neurological development and diseases. In *Nestin*-Cre mice, conditional KO of *Mettl3* in the nervous system causes transcriptome-wide dysregulation and premature death of cerebellar granule cells (CGCs), consequently leading to cerebellar hypoplasia [27]. Cortical-specific conditional KO of *Mettl3* and *Fto* in *Emx1*-Cre mice revealed that METTL3, but not FTO, is essential for the proper translation of crucial genes in cortical radial glial cells and intermediate progenitors [28]. By integrating single-cell RNA-seq (scRNA-seq) and MeRIP-seq, a recent study further explored the functions of METTL3 and m ^6^A in retinogenesis. Retinal progenitor cells (RPCs) and Müller glial cells are affected primarily in a retina-specific conditional knockout mouse model. The transition from RPCs to Müller glial cells is transcriptionally disrupted in the absence of m ^6^A, as RPC-specific transcripts fail to undergo timely degradation [286]. Overexpression of METTL3 also disrupts cerebellar development, with disorganized Purkinje and glial cells observed [29].

Similarly, depletion of *Mettl14* also leads to multiple defects in neurodevelopment. *Mettl14* knockout, and consequentially the loss of m ^6^A, in embryonic mouse brains dysregulates the degradation of neurogenesis-related transcripts, causing a prolonged cell cycle in radial glial cells and the extension of cortical neurogenesis into the postnatal stage [34]. A further study revealed that the m ^6^A reader FMRP, which mediates mRNA nuclear export in an m ^6^A-dependent manner, is key to this cell cycle regulation in neurogenesis [72]. *Mettl14*-deposited m ^6^A modification also promotes neural stem cell (NSC) self-renewal. *Mettl14* KO affects the NSC pool size, resulting in a lower number of late-born neurons during cortical neurogenesis. m ^6^A in NSCs regulates the decay of transcripts encoding histone-modifying enzymes and, in turn, regulates histone modification [35]. Disrupted cell cycle progression and differentiation were also observed in retinal progenitors upon *Mettl14* KO [36]. In addition to regulating mRNA metabolism, m ^6^A in rRNA, which is deposited by METTL5, is critical for myelination, intelligence and normal brain functions [49].

Although depletion of individual YTHDF readers does not cause complete penetrance of premature lethality in mice, potentially owing to functional redundancy, these m ^6^A readers remain critical regulators during neurogenesis. Depletion of all three YTHDF readers, but not individual readers, in the retina can recapitulate the phenotype of *Mettl14* KO, suggesting that they are the effectors of m ^6^A in retinogenesis with a certain level of functional redundancy [36]. Individual YTHDF readers also have unique functions. *Ythdf1*-KO mice also display learning and memory defects. YTHDF1 recognizes m ^6^A on a group of key genes in the hippocampal neurons, which enhances their translation in response to neuronal stimulation and contributes to hippocampal synaptic transmission and long-term potentiation [64]. *Ythdf2*-KO mice can only be retrieved at a sub-Mendelian ratio, and 80% of *Ythdf2* ^–/–^ mice are likely to die before weaning [67]. A closer look at those *Ythdf2* ^–/–^ mice revealed compromised neural development, and YTHDF2 is critical for the temporal regulation of neurodevelopmental-related transcript degradation [287]. Intriguingly, it has also been reported that conditional KO of *Ythdf2* in retinal ganglion cells (RGCs) results in improved visual acuity in mice, potentially due to increased RGC dendrite branching, which results in the formation of more synapses [288]. As mentioned in the previous section, the ability of PRRC2A to recognize m ^6^A was first reported in the context of oligodendrocyte specification and myelination. One of the targets of PRRC2A is *Olig2*, a key transcription factor that regulates the expression of myelin-associated genes in oligodendrocytes. The binding of PRRC2A to *Oligo2* transcripts in an m ^6^A-dependent manner promotes the stability of *Oligo2* transcripts and, in turn, contributes to oligodendrocyte specification [75].

The m ^6^A demethylases FTO and ALKBH5 are typically not deemed critical for neurodevelopment. Nevertheless, they may serve as a supporting mechanism in response to stress-induced conditions. For example, when exposed to hypobaric hypoxia, the absence of ALKBH5 leads to aberrant cell proliferation and differentiation within the cerebellum. Although the m ^6^A level is only modestly increased globally upon ALKBH5 deficiency, the m ^6^A level in a subset of cell fate regulatory transcripts is dysregulated. This dysregulation disrupts RNA metabolism, such as nuclear export, of these transcripts, leading to defective cerebellar development [29].

#### m ^5^C

The role of m ^5^C in neurodevelopment has been much less explored than the role of m ^6^A. Nevertheless, dysfunctions in m ^5^C writers, NSUN2 [87], NSUN5 [93] and NSUN6 [101], are associated with human neurological defects such as developmental delay and intellectual disability (ID). When *Drosophila* is used as a model, a lack of NSUN2 can lead to severe defects in short-term memory [87], and a lack of NSUN6 can impair locomotion and learning [101]. Furthermore, *Nsun2* deficiency in the mouse prefrontal cortex (PFC) was reported to cause substantial alterations in the neuronal translatome and glycine synthesis due to a deficiency in tRNA ^Gly^ isodecoders [289]. The deficiency of *Nsun5* is considered to contribute to WBS, a multisystem disease with severe neurological defects. *Nsun5*-KO mice also exhibit cognitive deficits [ 95– 97]. In fetal stages, NSUN5 is highly expressed in radial glial cells (RGCs) of the cerebral cortex. The depletion of *Nsun5* disrupts radial glial scaffolds and consequently the migration of neocortical neurons [96]. In the postnatal stages, NSUN5 is highly expressed in callosal oligodendrocyte precursor cells (OPCs) and oligodendrocytes (OLs), and *Nsun5*-deficient mice exhibit suppressed proliferation and a substantial reduction in these cell types. The protein levels of cell cycle-related genes are also reduced in the corpus callosum of *Nsun5*-deficient mice [95]. Owing to its vital role in ensuring the decoding ability of mt-tRNA ^Met^, NSUN3 is specifically associated with encephalomyopathy [ 16, 89]. Overall, these results highlight the importance of m ^5^C in neurodevelopment, which warrants further investigation.

### Cardiohepatic system

Although the detailed functions of RNA modifications in cardiovascular development remain largely unexplored, the m ^6^A writer METTL3 and reader YTHDC1, as well as the m ^7^G writer METTL1, are suggested to be important for normal cardiovascular development and functions. Cardiac-specific overexpression and deletion of METTL3, resulting in increased and reduced m ^6^A levels, respectively, both affect cardiomyocytes, albeit at different stages. METTL3 overexpression compensated for cardiac hypertrophy, but no functional defects were detected even under stress conditions. In contrast, no abnormalities were observed in the hearts of newborn *Mettl3*-cKO mice, suggesting that METTL3 is dispensable for postnatal heart development. However, *Mettl3*-cKO mice exhibit stress- and age-related cardiac failure, suggesting that METTL3 plays a critical role in adaptive cardiac remodeling after injury [30]. YTHDC1, but not other YTH family readers, is related to dilated cardiomyopathy (DCM). Combining MeRIP-seq, RIP-seq of YTHDC1 and mRNA-seq revealed that correct splicing of *Titin* (TTN), the most commonly known genetic cause of dilated cardiomyopathy [62], is regulated by YTHDC1, likely in a m ^6^A-dependent manner [290]. WTAP, a key component of the m ^6^A writer complex, is also linked to dilated cardiomyopathy. Intriguingly, WTAP deficiency results in reduced chromatin accessibility and transcriptional downregulation of *Mef2a* and *Mef2c*, which are key transcription factors in cardiomyocytes. However, whether this chromatin regulation of WTAP is m ^6^A-dependent remains to be confirmed [37].

Unlike that of m ^6^A-related proteins, the function of the m ^7^G writer METTL1 has not been investigated in cardiomyocytes but rather in cardiac fibroblasts. Defects in the regulation and functions of cardiac fibroblasts are often linked to cardiac fibrosis. A recent study identified METTL1 as a profibrotic factor and a potential therapeutic target in cardiac fibrosis. Enhanced METTL1-deposited m ^7^G was observed in cardiac fibrosis tissues, and *METTL1* KO ameliorated cardiac fibrosis, potentially due to the reduced m ^7^G level and translation of fibrotic genes [127].

RNA modifications undoubtedly also play critical roles in regulating hepatogenesis and maintaining normal liver function. For example, the Ψ writer DKC1 is essential for rRNA processing and the proliferation of hepatocytes [162]. However, the detailed molecular mechanisms involved have recently been revealed for only m ^6^A. Hepatic-specific KO of *Mettl3* causes perinatal hepatocyte injury, progenitor cell activation, and fibrosis, which eventually leads to postnatal lethality in mice. A master transcription factor of liver development, *Hnf4a*, is stabilized at the transcript level via METTL3-deposited m ^6^A and its reader IGF2BP1 [31]. Another group has also investigated the effect of hepatic-specific *Mettl3* KO. Despite some discrepancy with earlier studies, they revealed an additional METTL3-IGF2BP2-GYS2 axis, where IGF2BP2 recognizes METTL3-deposited m ^6^A and stabilizes the *Gys2* transcript, which encodes live glycogen synthase and is key to liver glycogenesis [32].

### Musculoskeletal system

#### Skeletal myogenesis

Skeletal muscle, which comprises a large number of myofibers, represents 30–40% of the human body mass. The process in which myogenic progenitor cells proliferate, differentiate and fuse to form multinucleated myofibers is termed myogenesis. Myogenesis occurs in both the embryonic and adult stages, as muscle stem cells, or satellite cells, reside at the periphery of adult myofibers and can be activated in response to injury, ensuring the robust regenerative capacity of muscle tissue. The proliferation and differentiation of muscle stem cells are highly related to their mitochondrial functions [291]. The m ^6^A eraser FTO and the Ψ writer PUS1 were reported to be essential for mitochondrial functions in muscle. FTO is required for myogenic differentiation. Depletion of FTO leads to reduced mitochondrial content, reduced expression of mtDNA-encoding genes and decreased ATP level. The expression of PGC-1α, a key regulator of mitochondrial biogenesis and dynamics, is also downregulated in the absence of FTO [53]. Mutation of PUS1 is linked to mitochondrial myopathy with lactic acidosis and sideroblastic anaemia (MLASA), an oxidative phosphorylation disease in humans. *Pus1*-KO mice show reduced exercise capacity at 14 weeks of age, accompanied by dysregulated muscle metabolism, reduced mitochondrial content and impaired oxidative capacity [155]. In addition, *Nsun5*, which installs m ^5^C on rRNA and is key for translation, also contributes to proper myogenesis. As mentioned above, NSUN5 has been linked to WBS, a multisystem disorder that is also characterized by decreased muscle mass [292]. Similarly, *Nsun5*-KO mice also exhibit decreased lean mass without changes in food intake [93].

#### Osteogenesis

Osteogenesis, or bone development, is a process required for bone homeostasis and injury repair and therefore persists throughout adulthood. The balanced activities between two main cell types of bone, bone-forming osteoblasts and bone-resorbing osteoclasts, are vital for proper osteogenesis and the regeneration capacity of bone. In situations where osteoclast activity exceeds osteoblast activity, osteoporosis occurs. A recent study reported an important role of METTL3 in bone marrow mesenchymal stem cells (MSCs), which are progenitors of osteoblasts. Loss of function of *Mettl3* impairs the osteogenic differentiation potential of mesenchymal stem cells (MSCs), whereas overexpression of *Mettl3* in MSCs protects mice from estrogen deficiency-induced osteoporosis. METTL3 in MSCs is critical for the translation of *Pth1r* (parathyroid hormone receptor-1), the primary receptor for PTH, and PTH/PTH1R signaling promotes MSC differentiation into osteoblasts and subsequent osteoblast proliferation and differentiation [ 33, 293]. Overall, *Mettl3*-mediated m ^6^A is vital for cell differentiation, and more epitranscriptomic mechanisms in bone development and diseases have yet to be revealed.

## Perspectives

Current studies with advanced sequencing methods and genetically modified animal models have extensively expanded our understanding of RNA Modifications and their functions.

The dynamic epitranscirptome, which regulates all aspects of the RNA life cycle, contributes significantly to the intricate regulatory networks that govern developmental processes and cellular identity. However, there are a few technical challenges that hinder the further exploration of RNA modifications, and several areas where knowledge is currently lacking.

### Modifications beyond m ^6^A and coordination across modifications

As discussed in the previous sections, RNA metabolism is strictly regulated by multiple RNA modifications and a complex network of related proteins. However, the majority of studies have focused on the roles of m ^6^A, the most prevalent RNA modification of mRNAs, although other modifications, such as Ψ, are also critical regulators of RNA metabolism during development and hold great potential in RNA-based therapeutics. In addition, only a few studies have explored how these RNA modifications are spatiotemporally coordinated with one another. Recent advances in long-read sequencing technologies, such as the Oxford Nanopore, indicate that RNA modifications co-occur on mRNA molecules [ 294– 296]. The mechanisms behind the co-occurrence of modifications and their implications are still not understood, necessitating the exploration of multiple hypotheses. For example, co-occurrence could involve a series of RNA modifications acquired at different processing stages, representing the history of one RNA molecule. Crosstalk between different writers and readers may promote the sequential addition of modifications. It is also possible that the co-occurrence of multiple modifications may have synergistic effects. Deciphering the co-occurrence of modifications in RNA molecules remains a largely unexplored area in the field of epitranscriptomics. In addition, the spatiotemporal specificity of RNA modification-related proteins is also an intriguing yet largely unexplored area.

### Interplay with additional non-coding RNAs

While current studies have focused primarily on the functions of RNA modifications in protein-coding mRNAs, emerging evidence suggests that modifications of ncRNAs—including lncRNAs, microRNAs, carRNAs, and retrotransposon transcripts—are also functional. Compared with protein-coding mRNAs, the metabolism of these ncRNAs is equally crucial, and understanding the interplay between RNA modifications and ncRNAs is essential for comprehensively dissecting epitranscriptomic regulation in development and disease.

### Deciphering modification stoichiometry

While sequencing methods with single-base resolution are available for several modifications, the required input quantity may still exceed what is feasible for rare cell types such as oocytes and early embryos. Furthermore, discrepancies regarding the outcomes of RNA modification profiling occasionally arise among studies. These discrepancies may stem from the varying biological contexts as well as the different sequencing methods applied. Therefore, precise measurement of the stoichiometry of RNA modifications, especially in rare cell types, remains a challenge. In addition to sequencing methods, CRISPR-based editing systems, which allow site-specific manipulation of RNA modifications [ 297, 298], hold great promise for elucidating the biological significance of RNA modification stoichiometry and for serving as therapeutic tools to modify disease-related sites.

### Clinical application of RNA modifications

RNA modifications possess significant potential for advancing disease diagnosis and targeted therapy. The use of the aberrant epitranscriptome as a diagnostic biomarker has been proposed for neurological disease [299] and cancer [300], and the targeting of RNA-modifying enzymes and readers has also been adopted as a therapeutic strategy [ 301, 302]. In addition, RNA modifications that affect RNA metabolism could be applied to modulate the stability and/or efficacy of RNA-based therapeutics. One well-known example is the contribution of Ψ and m ^1^Ψ modifications to the successful development of mRNA vaccines against coronavirus disease 2019 (COVID-19). These two modifications may prevent ribosome stalling and premature translation termination [ 234, 303], as well as ablate the immunogenicity of *in vitro* transcription (IVT) RNA [ 304 , 305]. Nevertheless, RNA modification-based therapeutics are still in their infancy. Further application of RNA modifications in therapeutics requires accurate profiling of the epitranscriptome in both physiological and pathological states, as well as a comprehensive understanding of their regulatory networks.

In summary, RNA modifications represent a layer of regulatory complexity, shaping gene expression programs and the cellular state during development and disease. The integration of multidisciplinary approaches, including high-throughput omics technologies, long-read sequencing and CRISPR-based editing tools, will be instrumental in deciphering the functional significance of RNA modifications and their implications in human health and disease.
